# Passive Localization of Mixed Far-Field and Near-Field Sources without Estimating the Number of Sources

**DOI:** 10.3390/s150203834

**Published:** 2015-02-06

**Authors:** Jian Xie, Haihong Tao, Xuan Rao, Jia Su

**Affiliations:** National Key Laboratory for Radar Signal Processing, School of Electronic Engineering, Xidian University, No.2 Taibai South Road, Xi'an 710071, China; E-Mails: xiejian1986@gmail.com (J.X.); raoxuancom@163.com (X.R.); hmlh@guet.edu.cn (J.S.)

**Keywords:** sensor array signal processing, DOA estimation, far-field, near-field, source localization, range estimation, fourth-order cumulants

## Abstract

This paper presents a novel algorithm for the localization of mixed far-field sources (FFSs) and near-field sources (NFSs) without estimating the source number. Firstly, the algorithm decouples the direction-of-arrival (DOA) estimation from the range estimation by exploiting fourth-order spatial-temporal cumulants of the observed data. Based on the joint diagonalization structure of multiple spatial-temporal cumulant matrices, a new one-dimensional (1-D) spatial spectrum function is derived to generate the DOA estimates of both FFSs and NFSs. Then, the FFSs and NFSs are identified and the range parameters of NFSs are determined via beamforming technique. Compared with traditional mixed sources localization algorithms, the proposed algorithm avoids the performance deterioration induced by erroneous source number estimation. Furthermore, it has a higher resolution capability and improves the estimation accuracy. Computer simulations are implemented to verify the effectiveness of the proposed algorithm.

## 1. Introduction

Source localization has received considerable attention in sensor array signal processing over the past decades [1]. Most of the existing algorithms concentrate on far-field sources (FFSs), whose wavefronts are plane waves. Many high resolution algorithms have been proposed for the direction-of-arrival (DOA) estimation under the far-field assumption, such as the multiple signal classification (MUSIC) method [2], the estimation of signal parameters *via* rotational invariance technique (ESPRIT) [3], and their derivatives [4–6]. However, in many practical applications, the radiating sources may lie in the Fresnel region of the array [7], which is defined as [0.62(*D*^2^/*λ*)^½^, 2*D*^2^/*λ*] where λ is the wavelength of the sources and *D* symbolizes the array aperture. In this region, the spherical wavefronts are characterized by both DOA and range parameters [8]. Consequently, traditional FFS DOA estimation algorithms are no longer applicable for near-field sources (NFSs) localization. Fortunately, many advanced algorithms have been proposed for NFSs localization, including the 2-D MUSIC algorithm [8], the covariance approximation (CA) method [9,10], the weighted linear prediction method [11], and the rank-reduction (RARE) type algorithms [12–14].

Moreover, both FFSs and NFSs may coexist in many situations of interest, such as seismic exploration, electronic surveillance and speaker localization using microphone arrays. Most of the algorithms which deal with pure FFSs or pure NFSs, may fail in the scenarios of mixed sources. Accordingly, there has been an increasing interest in mixed sources localization. A two-stage MUSIC (TSMUSIC) algorithm [15] is firstly developed to localize mixed FFSs and NFSs. Based on fourth order cumulants, the TSMUSIC algorithm can successfully estimate the parameters of mixed sources. However, its computational complexity is high due to the construction of high order cumulant matrices. To relieve the computational burden, an efficient oblique projection MUSIC (OPMUSIC) algorithm is proposed in [16], which only utilizes second-order statistics. However, this algorithm suffers from severe array aperture loss. In [17], a low-complexity ESPRIT algorithm is advanced to locate the mixed far-field and near-field cyclostationary sources. Yet, array aperture loss and reduced range estimation accuracy are two slight limitations of this method. In [18], the authors extend the array aperture by utilizing a special nested sparse linear array, which can improve the estimation accuracy. Unfortunately, the range estimation suffers from spurious peaks problem in this scheme. Based on the generalized ESPRIT (GESPRIT) algorithm [19], many algorithms have been proposed to localize mixed sources [20–23]. In [22], the related far-field components are eliminated from the signal subspace to improve the estimation accuracy. But its far-field component elimination technique would bring extra estimation errors. A covariance differencing method is proposed in [20,21] to generate more reasonable classification of the source types. Based on ESPRIT-Like and polynomial rooting, an efficient mixed sources localization algorithm is presented in [23]. However, for these GESPRIT-based algorithms, the number of NFSs they can resolve is less than half of the number of array sensors [24]. In [25–27], the authors utilize the sparse signal recovery technique for mixed sources localization, which provide improved estimation accuracy. The algorithms in [26,27] are based on the construction of fourth order cumulant matrices and vectors, whereas the anti-diagonal elements of the second-order array covariance matrix is exploited in [25]. However, these sparse-recovery-based algorithms call for an enormous amount of computations. Besides, the regularization parameter that balances the tradeoff between Frobenius norm and *ℓ*_1_ norm is difficult to determine.

Note that all of the previous algorithms are based on linear array configurations which assume that the array sensors are placed along the *y*-axis and the sources are located in the *y*-*z* plane. Therefore, only two-dimensional (2-D) parameters (elevation angles and ranges) need to be estimated. Recently, some methods are developed to localize mixed sources in the three-dimensional (3-D) space, which is characterized by range, azimuth angle and elevation angle. For these algorithms, the geometric structure of the array is critical for the localization performance. Based on a cross array, the authors in [28] have decoupled the 3-D parameters estimation problem into one-dimensional (1-D) spectral functions in three stages. Also, a separated steering vector-based algorithm is proposed in [29] using cross array configuration. In [30], spherical microphone arrays are utilized for 3-D localization. By exploiting the recurrent relation between spherical harmonics, the DOAs are obtained from ESPRIT-like approach and the ranges are determined by 1-D MUSIC spectral search.

However, all of the abovementioned methods are based on subspace decomposition, which requires exact determination of the source number. Although some designed criteria, such as the Akaike information criterion (AIC) [31] and the minimum description length (MDL) detection criterion [32], can be used to estimate the source number, they tend to generate erroneous estimates of the number of sources under the condition of low signal-to-noise ratio (SNR) and small sample size [33]. Furthermore, most of these methods need that both the number of FFSs and the number of NFSs are known or correctly estimated, which is an even more difficult task in practical applications. If the source enumeration of the FFSs or the NFSs is incorrect, these subspace based methods will suffer from performance deterioration induced by underestimation as well as overestimation of the subspace dimension.

In view of the previous analyses, the mixed sources localization problem is faced with the following main difficulties: (1) being able to localize mixed sources successfully with unknown source number; (2) reasonable classification of FFSs and NFSs; (3) avoiding multidimensional search; (4) alleviating aperture loss; (5) avoiding parameter matching.

Aiming at addressing the abovementioned difficulties, we propose in this paper a novel localization algorithm for mixed FFSs and NFSs. Firstly, we construct multiple fourth-order spatial-temporal cumulant matrices, which only depend on the DOA information. Based on the joint diagonalization structure of these matrices, a novel 1-D spatial spectrum function is derived to generate the DOA estimates of both FFSs and NFSs. Then, with the DOA estimates, the range parameters are obtained and automatically paired via beamforming technique. Also, the types the sources are reasonably identified. Compared with the traditional methods, the proposed one does not require the knowledge of the source number, and therefore prevents the performance deterioration induced by erroneous source enumeration. In addition, our method avoids multidimensional spectral search and aperture loss. Moreover, both the temporal and spatial structures of the observed data are utilized in our method, which also contribute to the improvement of resolution ability and estimation accuracy. Due to the linear array configuration, our algorithm is limited to localize mixed sources only in the 2-D domain (elevation angle and range). To address the azimuth-elevation-range (3-D) localization problem, we can extend the proposed algorithm by applying planar arrays, such as cross array, rectangular array and circular array.

The rest of the paper is organized as follows: Section 2 introduces the signal model of mixed FFSs and NFSs. The proposed method is described in Section 3. In Section 4, we reveal a relationship between TSMUSIC and the proposed method. Section 5 presents a comparison among TSMUIC, OPMUSIC and the proposed algorithm. In Section 6, simulations are conducted to validate the performance of our method. We conclude this paper in Section 7.

Throughout the paper, the complex conjugate, transpose, Hermitian transpose, pseudo inverse are denoted by (•)*, (•)*^T^*, (•)*^H^* and (•)^†^, respectively. **I**_*m*_ represents a *m* × *m* identity matrix, and **0**_*m,n*_ is a *m* × *n* zero matrix.

## 2. Problem Formulation

**Figure 1.**
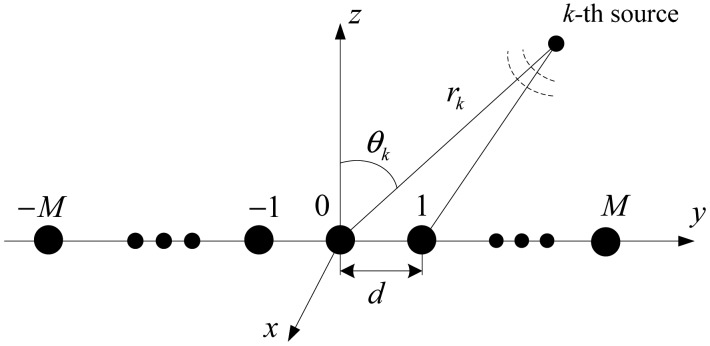
Symmetric uniform linear sensor array (ULSA) configuration for the proposed algorithm.

Consider *K* independent narrowband sources (near-field or far-field) sensed by a symmetric uniform linear sensor array (ULSA), which is composed of 2*M* + 1 omnidirectional sensors, as shown in Figure 1. All of these sensors are placed along the *y*-axis, with the inter-element spacing being *d*. Moreover, we assume that the impinging signals are in the *y*-*z* plane. As shown in Figure 1, the azimuth angles of impinging sources become 90° in this scenario, therefore, only the elevation angles and ranges need to be estimated. However, the proposed algorithm can be easily extended to address azimuth-elevation-range (3-D) localization problem by utilizing planar arrays or placing additional sensors along the x-axis.

The present signal model involves *K*_1_ sources in the far-field and the rest *K*_2_ sources in the near-field, where *K*_2_ = *K* − *K*_1_.

With the array center being the phase reference point, the data observed by the *m*th sensor at time index *t* has the following form:

(1) $$x_{m}\left(t\right)=\sum_{k=1}^{K}s_{k}\left(t\right)e^{j\tau_{mk}}+n_{m}\left(t\right)$$

where *s_k_*(*t*) is the *k*th source signal, *n_m_*(*t*) represents the *m*th sensor noise, *τ_mk_* is the phase shift associated with the *k*th source propagation time delay between the reference sensor and the *m*th sensor, which is of the form:

(2) $$\tau_{mk}=\frac{2\pi r_{k}}{\lambda }\left(\sqrt{1+{\left(\frac{md}{r_{k}}\right)}^{2}-\frac{2md\sin\theta_{k}}{r_{k}}}-1\right)$$

where *λ* is the source wavelength. It is obvious that the phase shift *τ_mk_* is a nonlinear function of the DOA parameter *θ_k_* and the range parameter *r_k_*. As a result, traditional FFS DOA estimation algorithms are no longer applicable. If the *k*th source is in the near-field, *τ_mk_* can be approximated as:

(3) $$\tau_{mk}\approx m\alpha_{k}+m^{2}\beta_{k}$$

herein, *α_k_* and *β_k_* are so-called electric angles given by [8]:

(4) $$\alpha_{k}=-\frac{2\pi d}{\lambda }\sin\theta_{k},\;\beta_{k}=\frac{\pi d^{2}}{\lambda r_{k}}\cos^{2}\theta_{k}$$

Note that, for the FFSs, the second term *β_k_* is approximated by zero and the associated range parameter *r_k_* is assumed to be ∞.

In a matrix form, the array output vector **x**(*t*) can be modeled as:

(5) $$\begin{array}{ll} x\left(t\right) & ={\left[x_{-M}\left(t\right),\cdots ,x_{0}\left(t\right),\cdots ,x_{M}\left(t\right)\right]}^{T}\\ & =\sum_{k=1}^{K}a\left(\theta_{k},r_{k}\right)s_{k}(t)+n\left(t\right)\\ & =\text{As}\left(t\right)+n\left(t\right) \end{array}$$

where **s**(*t*) is the *K* × 1 signal vector of the *K* sources:

(6) $$s\left(t\right)={\left[s_{1}(t),\cdots ,s_{k}(t),\cdots ,s_{K}(t)\right]}^{T}$$

**n**(*t*) is the (2*M* + 1)× 1 sensor noise vector:

**A** is the (2*M* + 1) × *K* steering matrix:

(8) $$n\left(t\right)={\left[n_{-M}\left(t\right),\cdots ,n_{0}\left(t\right),\cdots ,n_{M}\left(t\right)\right]}^{T}$$

with **a**(*θ_k_*,*r_k_*) being the steering vector of the *k*th source:

(9) $$a\left(\theta_{k},r_{k}\right)={\left[e^{j\left(-\alpha_{k}M+\beta_{k}M^{2}\right)},\cdots ,1,\cdots ,e^{j\left(\alpha_{k}M+\beta_{k}M^{2}\right)}\right]}^{T}$$

Given the observed array data, a novel algorithm is proposed in Section 3 to localize and distinguish the mixed sources successfully, under the following hypotheses.

(1) The DOA parameters *θ_k_*, *k* = 1, ⋯, *K* are distinct;
(2) The incoming signals are mutually independent, narrowband stationary, and non-Gaussian, having nonzero fourth-order cumulants;
(3) The noise is zero-mean, additive (white or color) Gaussian, and statistically independent from all impinging sources;
(4) In order to avoid the phase ambiguity, the inter-sensor spacing *d* should be within a quarter wavelength.

## 3. The Proposed Algorithm

### 3.1. Construction of the Spatial-Temporal Fourth-Order Cumulant Matrices

In this subsection, we apply the fourth-order cumulants to construct multiple spatial-temporal cumulant matrices. Due to the high degrees of freedom (DOF) available from cumulants, the resultant cumulant matrices are able to decouple the DOA estimation from the range estimation. Moreover, the array aperture size is fully utilized in these cumulant matrices so that the aperture loss problem is circumvented.

According to the definition in [34], at time lag *τ*, the fourth order spatial-temporal cumulant of the array outputs *x_m_*(*t* − *τ*),*x_n_*(*t*), *x_p_*(*t*),*x_q_*(*t*) can be written as:

(10) $$\begin{array}{l} \text{cum}\left\{x_{m}\left(t-\tau \right),x_{n}^{*}\left(t\right),x_{p}^{*}\left(t\right),x_{q}\left(t\right)\right\}\\ =\text{cum}\left\{\sum_{k=1}^{K}s_{k}\left(t-\tau \right)e^{j\left(m\alpha_{k}+m^{2}\beta_{k}\right)},\sum_{k=1}^{K}s_{k}^{*}\left(t\right)e^{-j\left(n\alpha_{k}+n^{2}\beta_{k}\right)},\sum_{k=1}^{K}s_{k}^{*}\left(t\right)e^{-j\left(p\alpha_{k}+p^{2}\beta_{k}\right)},\sum_{k=1}^{K}s_{k}\left(t\right)e^{j\left(q\alpha_{k}+q^{2}\beta_{k}\right)}\right\}\\ =\sum_{k=1}^{K}e^{j\left\{\left[\left(m+q\right)-\left(n+p\right)\right]\alpha_{k}+\left[\left(m^{2}+q^{2}\right)-\left(n^{2}+p^{2}\right)\right]\beta_{k}\right\}}\text{cum}\left\{s_{k}\left(t-\tau \right),s_{k}^{*}\left(t\right),s_{k}^{*}\left(t\right),s_{k}\left(t\right)\right\}\\ =\sum_{k=1}^{K}c_{4,sk}(\tau )e^{j\left\{\left[\left(m+q\right)-\left(n+p\right)\right]\alpha_{k}+\left[\left(m^{2}+q^{2}\right)-\left(n^{2}+p^{2}\right)\right]\beta_{k}\right\}} \end{array}$$

where *m*,*n*, *p*, *q* ∈ [−*M*,*M*], $c_{4,sk}(\tau )=\text{cum}\left\{s_{k}(t-\tau ),s_{k}^{*}(t),s_{k}^{*}(t),s_{k}(t)\right\}$ is the fourth-order cumulant of the *k*th source signal *s_k_*(*t*). For a given time lag *τ*, a (2*M* + 1)×(2*M* + 1) fourth-order cumulant matrix **C**_4*x*_ (*τ*) can be constructed, with its (*m* + *M* + 1, *n* + *M* + 1) th element being:

(11) $$c_{4,m,n}\left(\tau \right)=\text{cum}\left\{x_{m}\left(t-\tau \right),x_{-m}^{*}\left(t\right),x_{n}^{*}\left(t\right),x_{-n}\left(t\right)\right\}=\sum_{k=1}^{K}c_{4,sk}\left(\tau \right)e^{j2\left(m-n\right)\alpha_{k}}$$

Assuming that the source cumulants *c*_4,*sk*_ (*τ*) are nonzero for *L* different time lags *τ_l_*(1 ≤ *l* ≤ *L*), we can formulate *L* fourth-order spatial-temporal cumulant matrices, which can be expressed in a matrix form:

(12) $$C_{4x}\left(\tau_{l}\right)=\bar{A}C_{4s}\left(\tau_{l}\right){\bar{A}}^{H}=\sum_{k=1}^{K}c_{4,sk}\left(\tau_{l}\right)\bar{a}\left(\theta_{k}\right){\bar{a}}^{H}\left(\theta_{k}\right),l=1,\cdots ,L$$

where **C**_4*s*_(*τ_l_*) = *diag*[*c*_4,*s*1_(*τ_l_*), ⋯, *c*_4,*sK*_ (*τ_l_*)] is a *K* × *K* diagonal matrix, **Ā** = [**ā**(*θ*_1_), ⋯,**ā** (*θ_K_*)] is the (2*M*+1)×*K* virtual steering matrix, and **ā** (*θ_k_*) = [*e*^−*j*2*Mα_k_*^,*e*^−*j*2(*M* − 1)*α_k_*^, ⋯,1, ⋯,*e^j^*^2(^*^M^*^−1)^*^α_k_^*,*e^j^*^2^*^Mα_k_^*]*^T^* is the (2*M* + 1)×1 virtual steering vector. It is noteworthy that, in the cumulant domain, the observed NFSs are transformed into virtual FFSs, since the cumulant matrices **C**_4*x*_(*τ_l_*) (1 ≤ *l* ≤ *L*) only contain the DOA information.

### 3.2. DOA Estimation without Knowledge of Source Number

From Equation (12), it is evident that each of the *L* spatial-temporal cumulant matrices spans the same column space with that of the virtual steering matrix **Ā**. Therefore, with the joint diagonalization structure of the multiple cumulant matrices, we can utilize these matrices simultaneously to identify the column space of **Ā** and generate the DOA estimates of the mixed sources.

For the *k*th source, one can always find a vector **b**_*k*_ ∈ ℂ^2^*^M^*^+1^ which is orthogonal to the range space spanned by the virtual steering vectors except **ā**(*θ_k_*), *i.e.*:

(13) $$b_{k}⊥\text{span}\left\{\bar{a}\left(\theta_{1}\right),\cdots ,\bar{a}\left(\theta_{k-1}\right),\bar{a}\left(\theta_{k+1}\right),\cdots ,\bar{a}\left(\theta_{K}\right)\right\}$$

and the following notation can be equally used:

(14) $${\bar{a}}^{H}\left(\theta_{i}\right)b_{k}=\{\begin{array}{c} {\bar{a}}^{H}\left(\theta_{k}\right)b_{k}\\ 0 \end{array}\;\begin{array}{c} i=k\\ i\neq k \end{array}$$

Substituting Equation (14) into Equation (12) leads to:

(15) $$C_{4x}\left(\tau_{l}\right)b_{k}=\sum_{i=1}^{K}c_{4,si}\left(\tau_{l}\right)\bar{a}\left(\theta_{i}\right){\bar{a}}^{H}\left(\theta_{i}\right)b_{k}=d_{l}\bar{a}\left(\theta_{k}\right)$$

where *d_l_* = *c*_4,*sk*_ (*τ_l_*)**ā***^H^*(*θ_k_*)**b**_*k*_ is a scalar. This equation reveals that if the variable *θ* equals to one of the sources' DOAs, there always exists a scalar *d_l_* that makes **C**_4*x*_(*τ_l_*)**b** and **ā**(*θ*) colinear. Note that Equation (15) applies to all time lags *τ_l_*(1 ≤ *l* ≤ *L*). Consequently, we can transform the DOA estimation problem into the following optimization problem:

(16) $$\begin{array}{l} \underset{\theta }{\min}\;J\left(\theta ,b,d\right)=\sum_{l=1}^{L}{\left\|C_{4x}\left(\tau_{l}\right)b-d_{l}\bar{a}\left(\theta \right)\right\|}^{2}\\ \text{s}.\text{t}.\;\left\|d\right\|=1 \end{array}$$

where *θ* is the DOA parameter of interest, **b** ∈ ℂ^2^*^M^*^+1^ and **d** = [*d*_1_, ⋯,*d_L_*]*^T^*∈ ℂ*^L^* are the nuisance parameters. In order to avoid the trivial solution {**b**=**0, d**=**0**}, the constraint ∥**d**∥ = 1 is added.

From Equation (16), it is obvious that the computational complexity of this estimator is prohibitive when **b** and **d** are unknown. Therefore, to decrease the computational load, we first decouple the DOA parameter *θ* from other parameters, where only 1-D spectral search is required.

The objective function in Equation (16) can be expanded as:

(17) $$\begin{array}{c} J\left(\theta ,b,d\right)=b^{H}\left(\sum_{l=1}^{L}C_{4x}^{H}\left(\tau_{l}\right)C_{4x}\left(\tau_{l}\right)\right)b-b^{H}\left(\sum_{l=1}^{L}d_{l}C_{4x}^{H}\left(\tau_{l}\right)\bar{a}\left(\theta \right)\right)\\ -\left(\sum_{l=1}^{L}d_{l}^{*}{\bar{a}}^{H}\left(\theta \right)C_{4x}\left(\tau_{l}\right)\right)b+{\bar{a}}^{H}\left(\theta \right)\bar{a}\left(\theta \right)\sum_{l=1}^{L}{\left|d_{l}\right|}^{2} \end{array}$$

Let:

(18) $$C=\sum_{l=1}^{L}C_{4x}^{H}\left(\tau_{l}\right)C_{4x}\left(\tau_{l}\right)\in {\mathbb{C}}^{\left(2M+1\right)\times \left(2M+1\right)}$$

(19) $$Q\left(\theta \right)=\left[C_{4x}^{H}\left(\tau_{1}\right)\bar{a}\left(\theta \right),\cdots ,C_{4x}^{H}\left(\tau_{L}\right)\bar{a}\left(\theta \right)\right]\in {\mathbb{C}}^{\left(2M+1\right)\times L}$$

In view of that ${\sum_{l=1}^{L}d_{l}=\left\|d\right\|}^{2}=1$ and **ā***^H^*(*θ*) **ā** (*θ*)= 2*M* + 1, *J*(*θ*,**b**, **d**) in Equation (17) can be rewritten as:

(20) $$J\left(\theta ,b,d\right)=b^{H}Cb-b^{H}Q\left(\theta \right)d-d^{H}Q^{H}\left(\theta \right)b+2M+1$$

In order to decouple the nuisance parameter **b** from the objective function, we set the partial derivative of *J*(*θ*,**b**,**d**) with respect to **b** be zero, *i.e.*:

(21) $$\frac{\partial \;J\left(\theta ,b,d\right)}{\partial \;b}=2\left(Cb-Q\left(\theta \right)d\right)=0_{2M+1}$$

which yields:

(22) $$b_{\text{opt}}=C^{†}Q\left(\theta \right)d$$

Substituting Equation (22) into Equation (20), the optimization problem without the nuisance parameter **b** is of the following form:

(23) $$\begin{array}{l} \min\;J\left(\theta ,d\right)=2M+1-d^{H}Q^{H}\left(\theta \right)C^{†}Q\left(\theta \right)d\\ \text{s}.\text{t}.\left\|d\right\|=1 \end{array}$$

Apparently, the objective function in Equation (23) is equivalent to maximizing **d***^H^***Q***^H^* (*θ*)**C**^†^**Q**(*θ*)**d** with respect to **d**. Suppose the eigenvalue decomposition (EVD) of **Q***^H^* (*θ*)**C**^†^**Q**(*θ*) produces:

(24) $$Q^{H}\left(\theta \right)C^{†}Q\left(\theta \right)=\sum_{m=1}^{2M+1}\lambda_{m}v_{m}v_{m}^{H}$$

where *λ*_1_ ≥ ⋯≥ *λ*_2*M*+1_ are the eigenvalues arranged in the descend order, and **v**_*m*_, *m* = 1, ⋯,2M+1 are the corresponding eigenvectors. Accordingly, the maximum value of **d***^H^***Q***^H^*(*θ*)**C**^†^**Q**(*θ*)**d** is given by:

(25) $$\underset{\theta }{\max}\left\{d^{H}Q^{H}\left(\theta \right)C^{†}Q\left(\theta \right)d\right\}=\underset{\theta }{\max}\left\{\sum_{m=1}^{2M+1}\lambda_{m}d^{H}v_{m}v_{m}^{H}d\right\}=\underset{\theta }{\max}\left\{\sum_{m=1}^{2M+1}\lambda_{m}{\left\|d^{H}v_{m}\right\|}^{2}\right\}=\lambda_{1}$$

The last equation holds when **d** = **v**_1_ is the eigenvector corresponding to the maximum eigenvalue of **Q***^H^*(*θ*)**C**^†^**Q**(*θ*), which is *λ*_1_. Therefore, the estimation of DOA is further simplified as:

(26) $$\min\;J\left(\theta \right)=2M+1-\max\;\text{eig}\left(Q^{H}\left(\theta \right)C^{†}Q\left(\theta \right)\right)$$

Herein, ‘max eig’ stands for the maximum eigenvalue of a square matrix. Consequently, the DOA estimates of the mixed sources can be obtained from the highest peaks of the following 1-D spectral function:

(27) $$P\left(\theta \right)=\frac{1}{2M+1-\max\;\text{eig}\left(Q^{H}\left(\theta \right)C^{†}Q\left(\theta \right)\right)}$$

### 3.3. Range Estimation and Source Classification

In order to circumvent the source number enumeration, the minimum variance distortionless response (MVDR) beamformer [35,36] is utilized in this subsection for the range estimation problem. The output power spectrum of the MVDR beamformer is:

(28) $$P_{\text{MVDR}}\left(\theta ,r\right)=\frac{1}{a^{H}\left(\theta ,r\right)R^{-1}a\left(\theta ,r\right)}$$

where **R** = *E*{**x**(*t*)**x***^H^*(t)} is the second-order array covariance matrix. With the DOA estimates obtained in the previous subsection, the 2-D spectrum search over the DOA-range plane could be reduced to 1-D search on the range domain. Therefore, by substituting the estimated DOA *θ̂_k_* back into Equation (28), the range *r_k_* can be obtained as:

(29) $${\hat{r}}_{k}=\underset{r}{\max}\frac{1}{a^{H}\left({\hat{\theta }}_{k},r\right)R^{-1}a\left({\hat{\theta }}_{k},r\right)}$$

Since the NFSs lie in the Fresnel region, *r* is searched over [0.62(*D*^2^/*λ*)^½^, 2*D*^2^/*λ*]. If the estimation *r̂_k_* is in the Fresnel region, the corresponding source is identified as a near-field one; otherwise, it is determined to be in the far-field. Note that, in this procedure, the DOA estimation and range estimation are automatically paired without any additional operation.

### 3.4. Implementation of the Proposed Algorithm

Based on the above analyses, the implementation of the proposed algorithm is summarized as follows:

Step 1. Calculate the *L* fourth-order spatial-temporal matrices **C**_4*x*_(*τ_l_*),*l* = 1, ⋯, *L* according to Equation (11);
Step 2. Construct the matrices **C** and **Q**(*θ*) from Equations (18) and (19);
Step 3. Use Equation (27) to plot the DOA spectrum function *P*(*θ*), and find the DOA estimates;
Step 4. Find the range estimates using Equation (29), and classify the types of the sources;

## 4. Analogy between the Proposed Algorithm and TSMUSIC

In this subsection, we analyze the relationship between the proposed algorithm and TSMUSIC in the framework of subspace decomposition. If we take only one single spatial-temporal cumulant matrix **C**_4*x*_(*τ*_1_ = 0), the matrices **C** and **Q**(*θ*) in Equations (18) and (19) will be simplified as:

(30) $$C=C_{4x}^{H}\left(0\right)C_{4x}\left(0\right)$$

(31) $$Q\left(\theta \right)=C_{4x}^{H}\left(0\right)\bar{a}\left(\theta \right)$$

and consequently, **Q***^H^*(*θ*)**C**^†^**Q**(*θ*) can be reformulated as:

(32) $$Q^{H}\left(\theta \right)C^{†}Q\left(\theta \right)={\bar{a}}^{H}\left(\theta \right)C_{4x}\left(0\right){\left(C_{4x}^{H}\left(0\right)C_{4x}\left(0\right)\right)}^{†}C_{4x}^{H}\left(0\right)\bar{a}\left(\theta \right)={\bar{a}}^{H}\left(\theta \right)P_{C_{4x}}\bar{a}\left(\theta \right)$$

where $P_{C_{4x}}=C_{4x}\left(0\right){\left(C_{4x}^{H}\left(0\right)C_{4x}\left(0\right)\right)}^{†}C_{4x}^{H}\left(0\right)$ is the projection matrix that projects the vector in the vector space ℂ^2^*^M^*^+1^ onto the column space of **C**_4*x*_(0). Note that, **Q***^H^*(*θ*)**C**^†^**Q**(*θ*) is reduced to a scalar. Accordingly, the spectrum function in Equation (27) becomes:

(33) $$P\left(\theta \right)=\frac{1}{2M+1-{\bar{a}}^{H}\left(\theta \right)P_{C_{4x}}\bar{a}\left(\theta \right)}$$

The eigenvalue decomposition (EVD) of **C**_4*x*_(0) yields:

(34) $$C_{4x}\left(0\right)=E_{s}\Lambda_{s}E_{s}^{H}+\sigma^{2}E_{n}E_{n}^{H}$$

where **Λ**_*s*_ is the diagonal matrix containing *K* large eigenvalues. **E**_*s*_ is the (2*M* + 1) × *K* signal subspace eigenvector matrix of **C**_4*x*_(0). **E**_*n*_ is the (2*M* + 1) × (2*M* + 1 − *K*) eigenvector matrix spanning the noise subspace of **C**_4*x*_(0). Based on the orthogonality between the virtual steering matrix **Ā** and the noise subspace, TSMUSIC has the following DOA spectrum function [15]:

(35) $$P_{\text{TSMUSIC}}\left(\theta \right)=\frac{1}{{\bar{a}}^{H}\left(\theta \right)E_{n}E_{n}^{H}\bar{a}\left(\theta \right)}$$

Since **C**_4*x*_(0) is computed using fourth-order cumulants, *σ*^2^ = 0 (cumulants suppress additive Gaussian noise). In this case, **C**_4*x*_(0) spans the same range space of **E**_*s*_, *i.e.*, span(**C**_4*x*_(0)) = span(**E**_*s*_). Consequently, the projection matrix **P_C_**_4*x*_ can be also formulated as:

(36) $$P_{C_{4x}}=E_{s}{\left(E_{s}^{H}E_{s}\right)}^{-1}E_{s}^{H}=E_{s}E_{s}^{H}$$

By substituting Equation (36) into Equation (33), the DOA spectrum function of the proposed algorithm becomes:

(37) $$\begin{array}{ll} P\left(\theta \right) & =\frac{1}{2M+1-{\bar{a}}^{H}\left(\theta \right)E_{s}E_{s}^{H}\bar{a}\left(\theta \right)}=\frac{1}{2M+1-{\bar{a}}^{H}\left(\theta \right)\left(I_{2M+1}-E_{n}E_{n}^{H}\right)\bar{a}\left(\theta \right)}\\ & =\frac{1}{{\bar{a}}^{H}\left(\theta \right)E_{n}E_{n}^{H}\bar{a}\left(\theta \right)} \end{array}$$

which is identical to Equation (35). Therefore, we come to an interesting conclusion that the DOA estimator for TSMUSIC is a special case of our method, if only one spatial-temporal cumulant matrix is utilized. However, our method does not require the estimation of the source number, which makes it practically attractive.

## 5. Discussion

In this section, we compare the proposed algorithm with two newly developed mixed sources localization algorithms: TSMUSIC [15] and OPMUSIC [16]. We discuss all of these three methods from the following aspects:

(1) Computational complexity: Concerning the computational complexity, we only compare the major multiplications involved in the construction of the statistical matrices, eigenvalue decomposition (EVD) and spectral search. The searching steps for the DOA parameter and the range parameter are denoted as *θ*_Δ_ and *r*_Δ_. Let *T* and 2*M* + 1 symbolize the snapshot number and sensor number, respectively. TSMUSIC requires computing two fourth-order matrices with dimension (2*M* + 1) × (2*M* + 1) and (4*M* + 1) × (4*M* + 1), performing EVD of the two matrices, and executing spectral search for DOA estimation. OPMUSIC involves constructing two second-order covariance matrices with dimension (2*M* + 1) × (2*M* + 1) and (*M* + 2) × (*M* + 2), performing EVD of the two matrices, and executing spectral search for DOA and range estimation. The proposed algorithm constructs *L* fourth order cumulant matrices **C**_4*x*_ (*τ_l_*), a second order covariance matrix **R**, **C** and **Q(***θ***)**, whose dimensions are (2*M* + 1) × (2*M* + 1), (2*M* + 1) × (2*M* + 1), (2*M* + 1) × (2*M* + 1) and (2*M* + 1) × *L*, respectively. In addition, it requires implementing the EVD of **R**, as well as performing spectral search for DOA and range estimation. The comparison results are listed in Table 1. Summing these three components, we can determine the major computational burden of TSMUSIC as *O*(9(2*M* + 1)^2^ *T* +9 (4*M* + 1)^2^ *T* + 4 / 3 (2*M* + 1)^3^ +4 / 3 (4*M* + 1)^3^ + 180 (2*M* + 1)^2^ / *θ*_Δ_). For OPMUSIC, the main complexity is *O*((2*M* + 1)^2^ *T* + (*M* + 2)^2^ *T* + 4 / 3 (2*M* + 1)^3^ +4 / 3 (*M* + 2)^3^ + 180 (2*M* + 1)^2^ / *θ*_Δ_) + *K*(2*D*^2^ / *λ* − 0.62(*D*^3^ / *λ*)^1/2^)(2*M* + 1)^2^ / *r*_Δ_), while that of the proposed algorithm needs about *O*(9*L*(2*M* + 1)^2^*T* + (2*M* + 1)^2^*T* + *L*(2*M* + 1)^3^ + *L*(*M* + 1)^2^ + 4 / 3(2*M* + 1)^3^ + 180(2*M* + 1)^2^ / *θ*_Δ_ + (2*M* + 1)^2^ *K*(2*D*^2^ / *λ* − 0.62(*D*^3^ / *λ*)^1/2^)/*r*_Δ_. Therefore, the computational complexity of the proposed algorithm is higher than that of TSMUSIC and OPMUSIC. However, it is important to note that the proposed algorithm does not require the knowledge of the source number, which is highly desirable for practical applications where detection of the source number is generally a very difficult task.
(2) Maximum number of resolvable sources: With an ULSA of 2*M* + 1 sensors, TSMUSIC can construct (2*M* + 1) × (2*M* + 1) -dimensional matrix. Based on the subspace theory, it needs at least one eigenvector of the constructed matrix to span the noise subspace. Therefore, TSMUSIC can localize up to 2*M* sources. Similarly, OPMUSIC can handle only *M* sources simultaneously due to the half aperture loss in overlapping operation. However, the proposed algorithm does not require subspace decomposition. It only needs to assume *K* ≤ 2*M* + 1 to ensure that there exists a nonzero vector **b**_*k*_ satisfying Equation (14). In other words, the maximum number of resolvable sources for the proposed algorithm is 2*M* + 1 with the same sensor array configuration. As a result, our method outperforms TSMUSIC and OPMUSIC in resolving more sources.

## 6. Simulation Results

In this section, several numerical simulations are conducted to validate the performance of the proposed algorithm relative to TSMUSIC [15] and OPMUSIC [16]. In the following simulations, we consider an symmetric ULSA composed of 2*M* + 1 = 9 (*M* = 4) elements with *d* = *λ*_/ 4_. The impinging signals are narrowband, noncoherent, equi-power and with the non-Gaussian form *s_k_* (*t*) = *e^jω_k_t^*. For the proposed algorithm, *L* = 5 spatial-temporal cumulant matrices at the first five time lags are considered. Moreover, it is always assumed that the source number is correctly estimated for TSMUSIC and OPMUSIC. The performance is measured in terms of spatial spectrum, probability of resolution (PR) and root mean-square error (RMSE). The PR is defined as the ratio between the number of successful resolution and the total number of Monte Carlo runs. If the DOA estimates of two sources *θ̂_k_*,*k* = 1,2 satisfy |*θ_k_* − *θ̂_k_*| < Δ / 2, they are said to be successfully resolved. Herein, Δ signifies the angular separation between the two sources. The RMSE is defined as:

(38) $$\text{RMSE}=\sqrt{\frac{1}{M_{C}}\sum_{n=1}^{M_{C}}{\left({\hat{y}}_{n,k}-y_{k}\right)}^{2}}$$

where *y_k_* stands for the parameters such as *θ_k_* and *r_k_*, *ŷ_n,k_* denotes the estimation of *y_k_* in the *n*th trial, and *M_C_* is the number of Monte Carlo runs.

**Figure 2.**
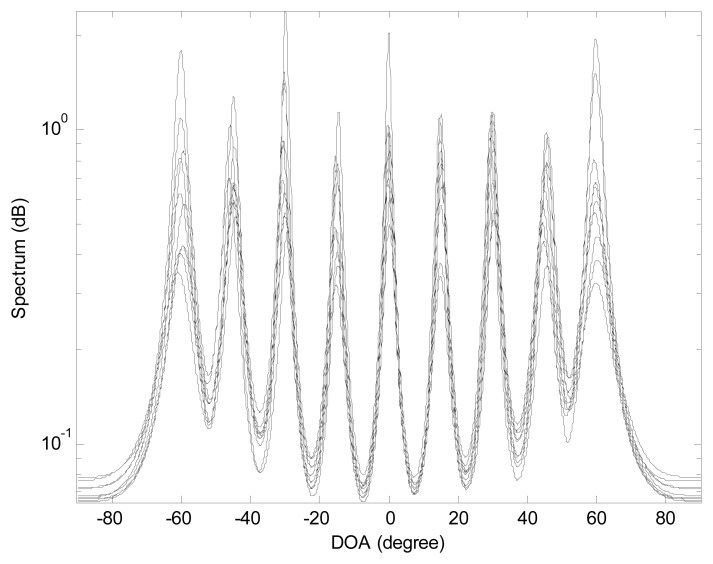
Mixed sources direction-of-arrival (DOA) spectra obtained with the proposed method. *K* = 9, 2*M* + 1 = 9. 10 independent trials are realized.

In the first experiment, we investigate the maximum number of resolvable sources for the proposed algorithm. We consider the mixed sources scenario that four FFSs are located at (*θ*_1_ = − 60°,*r*_1_ = + ∞), (*θ*_2_ = − 45°,*r*_2_ = + ∞), (*θ*_3_ = − 30°,*r*_3_ = + ∞), (*θ*_4_ = − 15°,*r*_4_ = + ∞), and five NFSs are placed at (*θ*_5_ = 0°,*r*_5_ = 0.5*λ*), (*θ*_6_ = 15°,*r*_6_ = 0.8*λ*), (*θ*_7_ = 30°,*r*_7_ = *λ*), (*θ*_8_ = 45°,*r*_8_ = 1.5*λ*), (*θ*_9_ = 60°,*r*_9_ = 2*λ*), respectively. The snapshot number and the SNR equal to 200 and 20 dB, respectively. Figure 2 indicates the DOA spectra from 10 independent realizations. It is evident that, with the 9-element ULSA, there are nine clearly discernible peaks corresponding to these mixed sources, which is in agreement with the analysis in Section 5.

**Figure 3.**
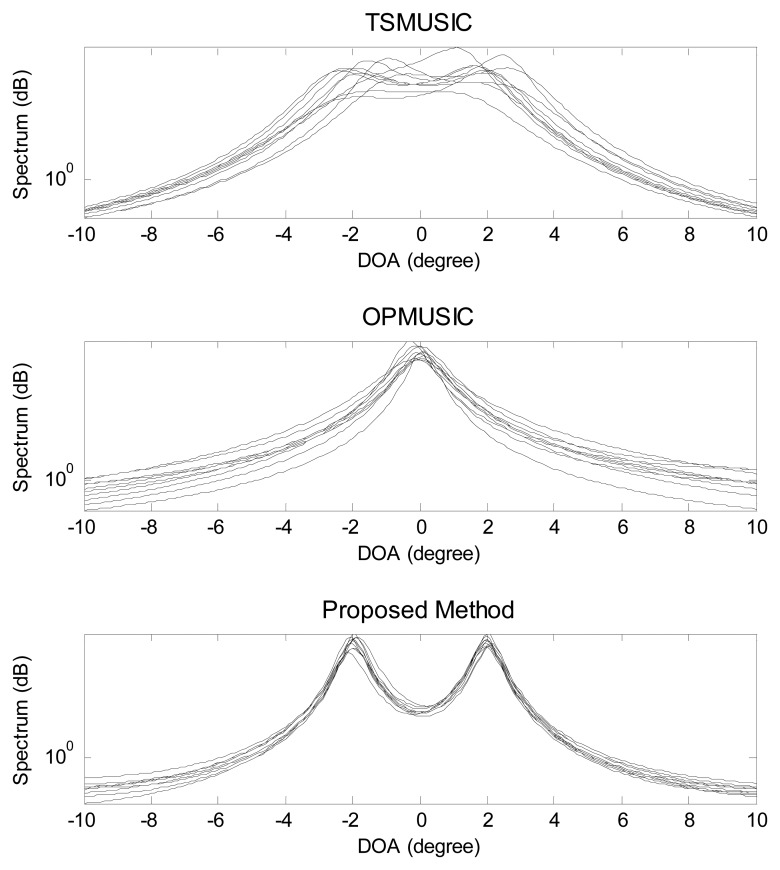
Mixed sources DOA spectra obtained with Two-Stage MUSIC (TSMUSIC), Oblique-Projection MUSIC (OPMUSIC) and the proposed method. 10 independent trials are realized for each method.

In the second experiment, the scenario of coexistence of one FFS and one NFS is investigated, with the location parameters being (*θ*_1_ = − 2°,*r*_1_ = 1.5*λ*) (NFS) and (*θ*_2_ = 2°,*r*_2_ = + ∞) (FFS). The snapshot number and the SNR equal to 500 and 5 dB, respectively. Ten independent trials of DOA spectrum estimates using the three methods are realized as is shown in Figure 3. It is obvious that, for the proposed method, there are two distinct peaks corresponding to the actual DOAs of the two mixed sources. However, TSMUSIC and OPMUSIC fails to distinguish the two closely spaced angles *θ*_1_ and *θ*_2_. This is because that, for closely spaced sources, it is very difficult for the subspace-based algorithms to correctly identify the signal subspace and noise subspace with low SNR, while the proposed method does not need any decomposition and identification of the subspaces.

**Figure 4.**
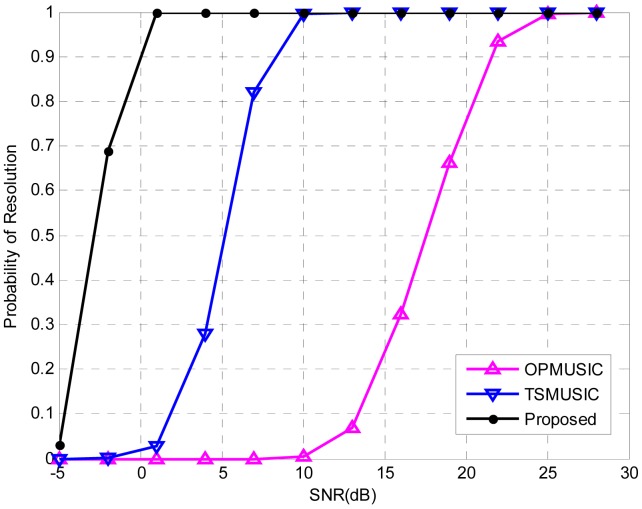
Probability of resolution (PR) versus signal-to-noise ratio (SNR). 500 independent trials are realized for each of the three methods.

In the third experiment, the PR of the three algorithms versus SNR is explored. Consider two uncorrelated equi-power sources locating at (*θ*_1_ = − 2°,*r*_1_ = 1.5*λ*) (NFS) and (*θ*_2_ = 2°,*r*_2_ = +∞) (FFS), with number of snapshots being 500. The SNR varies from −5 dB to 28 dB in steps of 3 dB. At each SNR, 500 independent Monte Carlo trials are performed. Figure 4 illustrates the PR of the three algorithms as a function of SNR. It is obvious that the proposed method has the best resolution ability since it reaches a full PR at the lowest SNR threshold (1dB). For TSMUSIC and OPMUSIC, the SNR thresholds of full PR are 10 dB and 25 dB, respectively, which are much higher than that of our method. This is due to the fact that TSMUSIC and OPMUSIC suffer from the leakage between the signal subspace and noise subspace when SNR is low, while the subspace decomposition is not required in our algorithm, which promotes the resolution ability at low SNRs. Moreover, OPMUSIC has the worst resolution ability since its DOA estimator has a half aperture loss, while the array size is fully utilized in TSMUSIC and our method.

**Figure 5.**
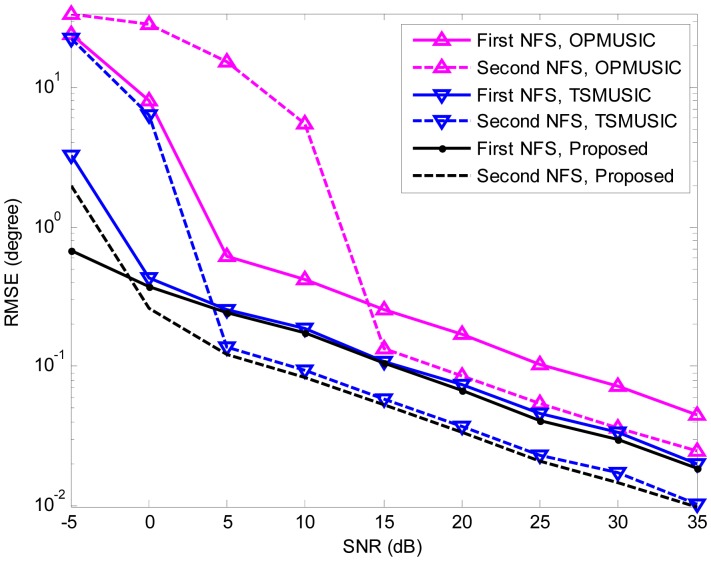
Root mean-square errors (RMSEs) of near-field sources (NFSs) DOA estimates *versus* SNR. 500 independent trials are realized for each of the three methods.

**Figure 6.**
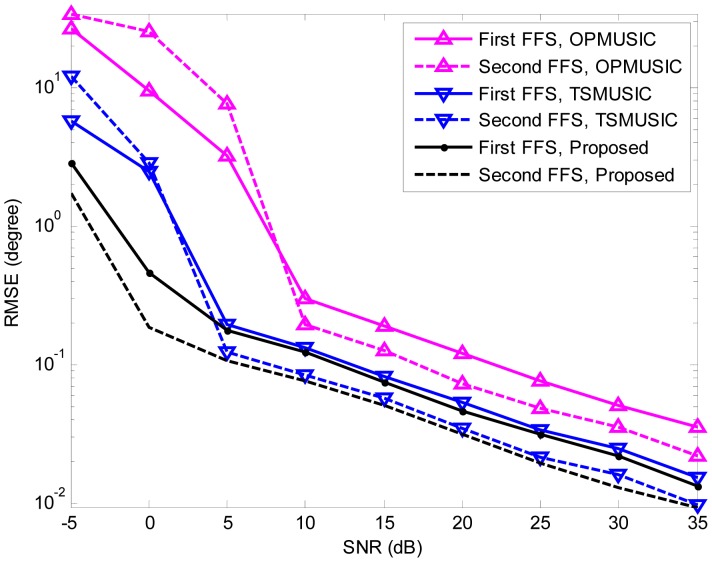
RMSEs of far-field sources (FFSs) DOA estimates *versus* SNR.

**Figure 7.**
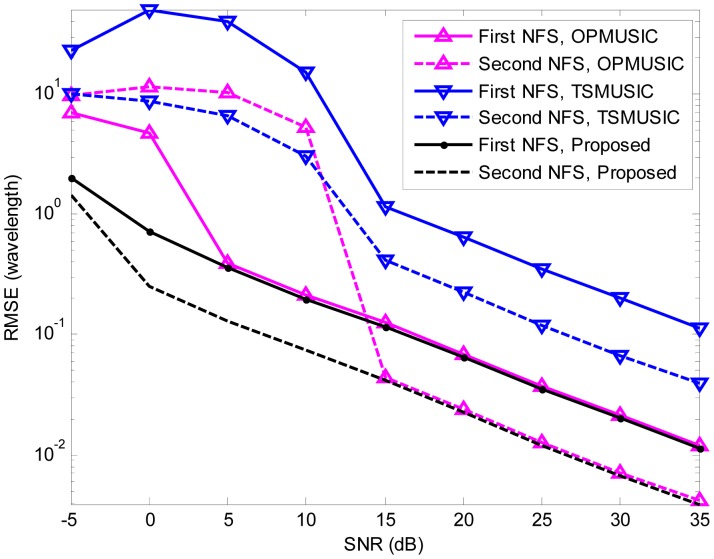
RMSEs of NFSs range estimates *versus* SNR.

In the fourth experiment, we study the estimation accuracy of the three algorithms as a function of SNR. A more general mixed sources scenario is considered, where two FFSs are located at (*θ*_1_ = 10°,*r*_1_ = +∞), (*θ*_2_ = 50°,*r*_2_ = +∞), and two NFSs are placed at (*θ*_3_ = − 10°,*r*_3_ = 1.5*λ*), (*θ*_4_ = − 30°,*r*_4_ = 3*λ*) (*θ*_4_ = − 30°,*r*_4_ = 3*λ*), respectively. The SNR varies from −5 dB to 35 dB in steps of 5 dB, and the snapshot number is fixed at 200. At each SNR, 500 independent Monte Carlo trials are performed. Figures 5, 6 and 7 illustrate the RMSEs of DOA and range estimates using the three algorithms. It can be seen that the estimation accuracy (both DOA and range) of the proposed algorithm outperforms that of TSMUSIC and OPMUSIC, especially in the low SNR region. This can be also explained from the fact that incorrect identifications of signal subspaces are likely occur at low SNRs, which would deteriorate the performance of TSMUSIC and OPMUSIC. At the high SNR region, the DOA estimation accuracy of TSMUSIC approaches to that of our method, which is in agreement with the analysis presented in Section 4. OPMUSIC has the worst DOA estimation accuracy because of its half aperture loss. As for the range estimation, in the low SNR region, the proposed algorithm is still superior to TSMUSIC and OPMUSIC, which is mainly a result of the propagation error from the previous DOA estimation stage. In the high SNR region, however, OPMUSIC and our method have almost the same performance. Additionally, TSMUSIC has the worst range estimation performance since it only utilizes the information of a single eigenvector with the smallest eigenvalue to obtain the range estimates. As is described in [15], the DOA estimates and the range estimates are obtained from two different stages in TSMUSIC. In the first stage, a special cumulant matrix is constructed and decomposed. DOAs can be obtained via high resolution MUSIC algorithm. In the second stage, another particular cumulant matrix is constructed to avoid the estimation failure problem. However, it only utilizes the information of a single eigenvector with the smallest eigenvalue to obtain the range estimates in this stage. Therefore, TSMUSIC has a good performance in bearing estimation but a worst performance in range estimation in the simulations.

**Figure 8.**
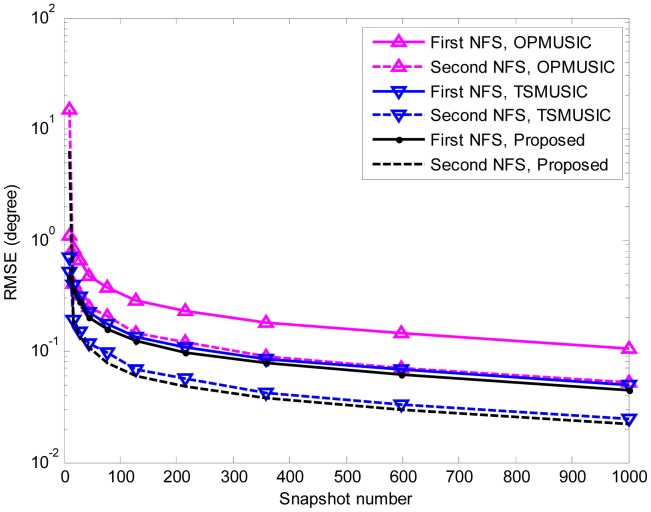
RMSEs of NFSs DOA estimates *versus* snapshot number. 500 independent trials are realized for each of the three methods.

**Figure 9.**
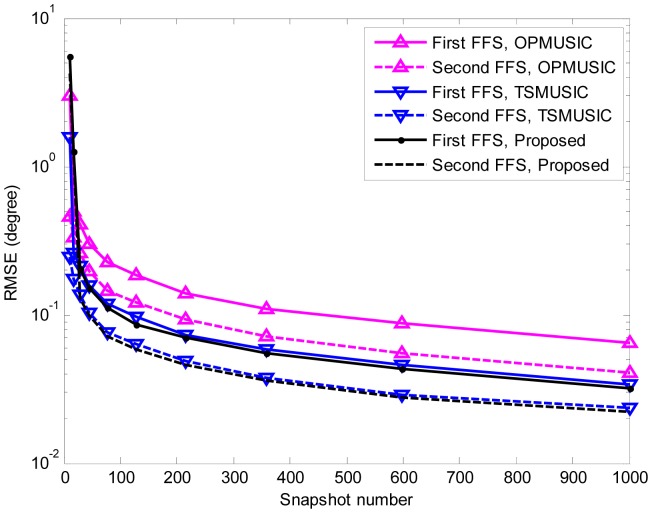
RMSEs of FFSs DOA estimates *versus* snapshot number. 500 independent trials are realized for each of the three methods.

**Figure 10.**
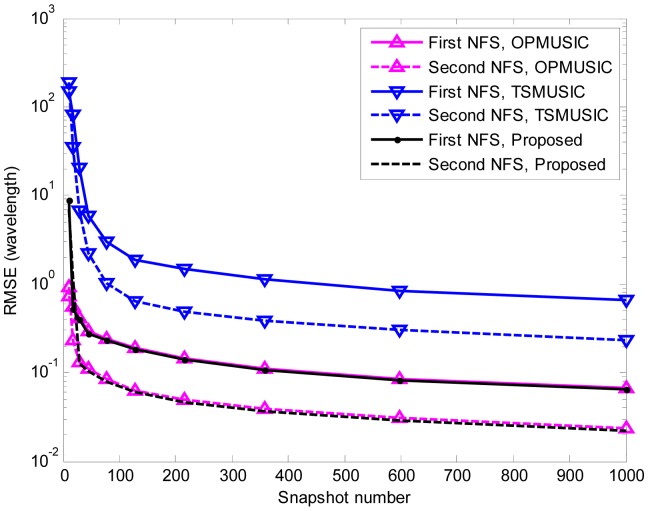
RMSEs of NFSs range estimates *versus* snapshot number. 500 independent trials are realized for each of the three methods.

In the fifth experiment, the same parameters as the third experiment are adopted except that the SNR is set equal to 15 dB, and the number of snapshots varies from 10 to 1000. At each snapshot number, 500 independent Monte Carlo trials are performed. The RMSEs of DOA and range estimation are shown in Figure 8, 9 and 10. It is obvious that the RMSEs (both DOA and range) of the three algorithms decrease monotonically as the snapshot number increases. This is due to the fact that a larger sampling number will produce better estimate of the cumulant matrices and the covariance matrices for stationary data. Furthermore, when the snapshot number is large enough, the proposed algorithm slightly outperforms TSMUSIC and OPMUSIC. This is because that: (1) the array aperture is fully utilized in this algorithm; (2) both the temporal and spatial structures of the observed data are utilized in our method, which also contribute to the improvement of estimation accuracy. However, when the sample size is small (*i.e.*, *snapshot number* = 10), the DOA estimation accuracy of our method is a little bit inferior to that of TSMUSIC and OPMUSIC. This can be explained that the estimation of the spatial-temporal cumulant matrices is not reliable when the sample size is insufficient.

**Figure 11.**
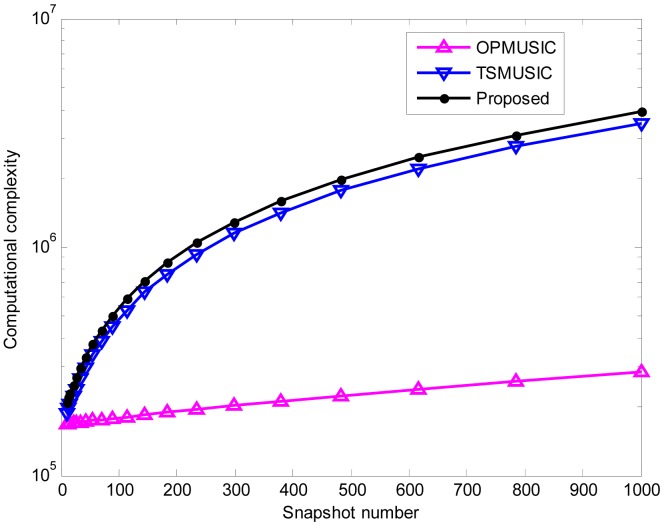
Computational complexity of the three methods *versus* snapshot number.

In the last experiment, we compare the computational complexity of the three algorithms. Suppose that there are 2*M* + 1 = 9 sensors, and that the searching steps *θ*_Δ_ and *r*_Δ_ are set as 0.1° and 0.05*λ*, respectively. The number of snapshots varies from 10 to 1000. Furthermore, we define *K* = 2 and *L* = 5. Figure 11 illustrates the computational burden of these algorithms as a function of the snapshot number. It is clear that the proposed algorithm has slightly higher complexity than TSMUSIC, while OPMUSIC is most efficient in computational complexity since it only involves second-order statistics.

## 7. Conclusions

In this paper, a novel algorithm has been proposed for the localization of mixed FFSs and NFSs. Based on the joint diagonalization structure of multiple fourth-order spatial-temporal cumulant matrices and the beamforming technique, the DOA and range parameters of FFSs and NFSs have been determined respectively. Compared with some existing mixed source localization methods, the new method has the main advantage that it does not require the information of the source number. Such an advantage is practically attractive since mixed source enumeration is typically a very difficult problem. In addition, we have found that our method is a generalization of the TSMUSIC algorithm in [15], which only utilize one single spatial-temporal cumulant matrix. Moreover, the proposed algorithm avoids multidimensional search, aperture loss and parameter matching. According to the simulation results, the proposed method outperforms TSMUSIC and OPMUSIC in both resolution ability and estimation accuracy, especially when the SNR is low.

It is worth mentioning that there are two slight limitations of the proposed algorithm. First, the construction of cumulant matrices leads to high computational load. Second, it is limited to localize mixed sources only in the 2-D domain (elevation angle and range). To put the proposed algorithm into further applications, several steps of the future work can be shown as follows:

1. To reduce the computational complexity, second-order statistics and real-valued transformation may be applied.
2. To extend the proposed algorithm for joint azimuth-elevation-range (3-D) estimation problem, we may employ planar arrays, such as cross array, rectangular array and circular array.

## Figures and Tables

**Table 1.** Computational complexity comparison.

**Algorithms**	**Statistical Matrices**	**EVDs**	**Spectral Search**
TSMUSIC	9(2*M* + 1)^2^ *T* + 9(4*M* + 1)^2^ *T*	4 / 3(2*M* + 1)^3^ + 4 / 3 (4*M* + 1)^3^	$\frac{180{\left(2M+1\right)}^{2}}{\theta_{\Delta }}$

OPMUSIC	(2*M* + 1)^2^ *T* +(*M* + 2)^2^ *T*	4 / 3(2*M* + 1)^3^ + 4 / 3(*M* + 2)^3^	$\begin{array}{l} K\cdot \frac{2D^{2}/\lambda -0.62{\left(D^{3}/\lambda \right)}^{1/2}}{r_{\Delta }}{\left(2M+1\right)}^{2}\\ +\frac{180{\left(2M+1\right)}^{2}}{\theta_{\Delta }} \end{array}$

Proposed Algorithm	9*L*(2*M* + 1)^2^ *T* +(2*M* + 1)^2^ *T* +*L*(2*M* + 1)^3^ + *L*(*M*+1)^2^	4 / 3(2*M* + 1)^3^	$\begin{array}{l} K\cdot \frac{2D^{2}/\lambda -0.62{\left(D^{3}/\lambda \right)}^{1/2}}{r_{\Delta }}{\left(2M+1\right)}^{2}\\ +\frac{180{\left(2M+1\right)}^{2}}{\theta_{\Delta }} \end{array}$
